# Plasma Biomarkers Can Predict Treatment Response in Tuberculosis Patients: A Prospective Observational Study

**DOI:** 10.1097/MD.0000000000001628

**Published:** 2015-10-02

**Authors:** Meng-Rui Lee, Chia-Jung Tsai, Wei-Jie Wang, Tzu-Yi Chuang, Chih-Mann Yang, Lih-Yu Chang, Ching-Kai Lin, Jann-Yuan Wang, Chin-Chong Shu, Li-Na Lee, Chong-Jen Yu

**Affiliations:** From the Department of Internal Medicine, National Taiwan University Hospital Hsin-Chu Branch, Hsin-Chu City, Taiwan (M-RL, L-YC, C-KL); Department of Internal Medicine, National Taiwan University Hospital, Taipei, Taiwan (M-RL, L-YC, C-KL, J-YW, L-NL, C-JY); Institute of Epidemiology and Preventive Medicine, College of Public Health, National Taiwan University (M-RL); Department of Internal Medicine, Taoyuan General Hospital, Taoyuan (C-JT, W-JW, T-YC); Department of Laboratory, National Taiwan University Hospital, Hsin-Chu Branch, Hsin-Chu (C-MY); Department of Traumatology (C-CS); and Department of Laboratory Medicine, National Taiwan University Hospital, Taipei, Taiwan (L-NL).

## Abstract

Despite numerous studies, there has been little progress in the use of biomarkers for predicting treatment response in patients with tuberculosis (TB).

Patients with culture-confirmed pulmonary TB between 2010 and 2014 were prospectively recruited. Blood samples were taken upon diagnosis and 2 months after the start of standard anti-TB treatment. A pilot study utilizing measurement of TB-antigen-stimulated cytokines was conducted to select potential biomarkers for further testing. Outcome was defined as persistent culture positivity at 2 months into treatment.

Of 167 enrolled patients, 26 had persistent culture positivity. RANTES, IL-22, MMP-8, IL-18, MIG, and Granzyme A were selected as potential biomarkers. For predicting persistent culture positivity, receiver-operating characteristics (ROC) analysis showed that initial RANTES (AUC: 0.725 [0.624–0.827]) and 2-month MMP-8 (AUC: 0.632 [0.512–0.713]) had good discriminative ability. Using a logistic regression model, low initial RANTES level (<440 pg/mL), initial smear positivity, and high 2-month MMP-8 level (>3000 pg/mL) were associated with persistent culture positivity. Low initial RANTES level and initial smear positivity had a positive predictive value of 60% (12/20) for persistent culture positivity, compared with 4% (3/75) among patients with high RANTES level and smear negativity upon diagnosis. In the 72 patients with either low RANTES/smear negativity or high RANTES/smear positivity upon diagnosis, the 2-month MMP-8 level had a positive and negative predictive value of 24 and 94%, respectively, for 2-month culture status.

Aside from an initial sputum smear status, serum RANTES level at diagnosis and MMP-8 level at 2 months of treatment may be used to stratify risk for culture persistence.

## INTRODUCTION

Tuberculosis (TB) is a global public health threat, accounting for 1.3 million deaths in HIV-negative population worldwide in 2013.^1^ In Taiwan, marked success in reducing the TB disease burden and incidence has been due to public health campaigns in recent years. The incidence and mortality rates both decreased, from 72.5 to 53 per 100,000 population and from 4.3 to 2.7 per 100,000 population, respectively, from 2005 to 2012.^2^ The World Health Organization (WHO) has now set new global targets for better TB control aimed at reducing TB deaths and incidence rate by 75% and 50%, respectively, by 2025.^3^ To achieve this, more accurate parameters predicting therapeutic response in the early stage of treatment are needed.

TB patients with greater disease extent and severity require longer treatment and are at higher risk for relapse following treatment.^4,5^ While no single clinical parameter before anti-TB treatment can reliably predict treatment response, sputum smear and culture status after 2 months of anti-TB medication are the most commonly used indicators.^6,7^ However, concerns exist that patients who do not respond to initial anti-TB treatment would experience clinical deterioration or develop drug resistance during this 2-month interval. These concerns lead to the hypothesis that host immunologic markers, either alone or in combination with clinical parameters, can be used to predict early treatment response.^8–10^ Thus, researchers in the past decade have been intensively searching for host immunologic markers that correlate with disease extent and may indicate a risk for unfavorable outcome at baseline.^11^ Most studies, however, are limited by a small patient number (usually <100 active TB patients) or only depict a trend of cytokine change.^9^ Clinical applications are also not readily available.

This study aimed to search for potential biomarkers that can aid in predicting culture positivity 2 months after the start of anti-TB treatment, and thereby also identify more predictive tools that can further stratify patient risk and optimize patient care.

## METHODS

### Study Design and Duration

This study was conducted at the National Taiwan University Hospital, a 2900-bed tertiary care center in northern Taiwan for the period 2010 to 2014. Its branch, the National Taiwan University Hospital Hsin-Chu Branch, has also been included in the process of recruitment since 2014. The Institutional Review Boards of both hospitals approved the study (NTUH REC: 95617008; 201108022RC; 201312097RIN; and NTUH-HC REC: 102-050-E).

The study consisted of 2 parts. First, a pilot study was conducted in 40 patients, including 10 with persistent culture positivity at 2-month and 30 with negative sputum culture at 2-month. The performance of cytokines in plasma and culture supernatant after in vitro stimulation with TB-specific antigens for the prediction of 2-month culture status was compared. Potential biomarkers were selected for further testing in the second part of the study.

### Study Population and Timing of Blood Sampling

**FIGURE 1:**
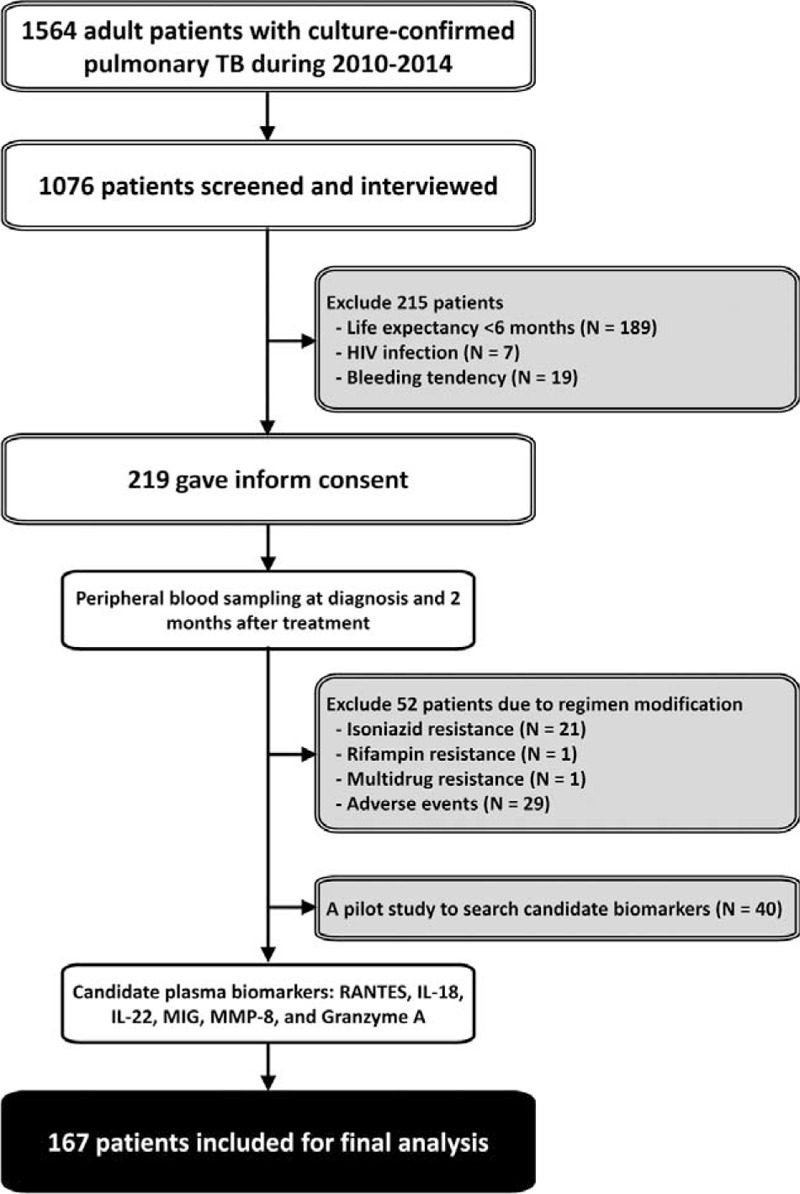
The patient recruitment process. IL = interleukin, MIG = monokine induced by gamma interferon, MMP = matrix metalloproteinase, RANTES = regulated on activation, normal T cell expressed, and secreted, TB = tuberculosis.

All adult patients (age >20 yr) with culture-confirmed TB were prospectively recruited. Participants were excluded if they met the exclusion criteria (Figure 1).

After providing informed consent, the patients underwent peripheral blood sampling upon diagnosis and 2 months after the start of anti-TB treatment for measurement of plasma levels of biomarkers. Patients with 2-month sputum samples that remained culture-positive for *Mycobacterium tuberculosis* were considered persistent culture positivity.

### Mycobacteriology Testing

All respiratory specimens sent for mycobacterial culture were processed as previously described.^12,13^ Mycobacterial species were identified by biochemical testing.^14^ Quality control assessment of National Taiwan University Hospital mycobacterial laboratory was periodically performed by the National Reference Laboratory of the Centers for Disease Control of Taiwan.^15^

### In vitro Stimulation of Peripheral Blood

In vitro stimulation of peripheral blood was performed using QuantiFERON-TB Gold In-Tube assay (QFT, Cellestis, Australia) according to the manufacturer's instructions. A 3-tube system of QFT was used, including the negative control tube (NC tube), positive control tube (PC tube), and TB-antigen tube (AG tube). After overnight culture, the supernatant of each tube was collected for biomarker measurement.

### Measurements of Biomarkers

Inflammatory and apoptosis markers in culture supernatant and plasma were assayed by using the Bioplex Multiplex Suspension Array System (Bio-Rad Laboratories Taiwan Ltd., Taipei City, Taiwan, R.O.C.) and enzyme-linked immunosorbent assay (ELISA).^16,17^ A systemic review was first conducted to identify potential cytokines. Interleukin (IL)-6, IL-10, IL-12, IL-17, IL-18, IL-22, IL-23, IP-10 (CXCL10, interferon gamma-induced protein 10), RANTES (CCL5, regulated on activation, normal T cell expressed and secreted), MIG (CXCL9, monokine induced by gamma interferon), and MCP-1 (monocyte chemoattractant protein) were reported to be important immune cytokines involved in TB.^9,18–22^ Being important mediators of the cytolytic pathway, Perforin, Granzyme A, Granzyme B, and soluble Fas ligand (sFasL) were also included.^23^ Known for their role in granuloma formation and tissue destruction in TB, metalloproteinases (MMPs) including MMP-1, MMP-3, MMP-8, MMP-9, and MMP-12 were also chosen as candidate biomarkers.^24^

### Data Collection

We collected demographic and clinical data with a prespecified case record form. We used radiographic severity (RS) score to assess disease image extent and the judgement of RS has been described in detail in previous studies.^13,25^

### Statistical Analysis

To describe the demographic, clinical, and radiographic characteristics, we used proportions or means. For the comparison between continuous variables, we used independent sampled *t* test, and between categorical variables, we used *χ*^2^ test.^13,25^ The discriminative power of each biomarker for 2-month culture status was analyzed using the receiver-operating characteristic (ROC) curve and area under the ROC curve (AUC). The optimal cut-off value was set according to Youden's index, which depended on the maximized value of sensitivity plus specificity minus 1.^26^

Logistic regression analysis was used to identify factors associated with persistent culture positivity after 2 months of anti-TB treatment. Cytokine levels were transformed into binary variables according to Youden's index before being entered into the logistic regression analysis. All of the potential predictors were included in the stepwise variable selection process. A 2-sided *P* < 0.05 was considered significant. All analyses were performed using the SPSS v13.0 (SPSS Inc, Chicago, Illinois). Graphics were drawn with Stata version 11 (StataCorp, College Station, Texas).

## RESULTS

### Patient Recruitment and Cytokines Selection

On the basis of the patient recruitment process (Figure 1), 219 patients were recruited from 1564 adult patients with culture-confirmed pulmonary TB during the period 2010 to 2014. Among them, 52 had either drug-resistant *M. tuberculosis* strain or experienced drug-related side-effects necessitating suspension or modification of anti-TB regimen during the first 2 months, and were therefore excluded. A total of 167 patients were included in the analysis.

### Pilot Study for Potential Biomarkers

Forty patients were selected for the pilot study to identify potential biomarkers predicting persistent culture positivity. RANTES, MIG, IL-18, IL-22, MMP-1, and MMP-8 level in the QFT culture supernatant had the potential to differentiate between 2-month culture status (all *P* < 0.01) (Table 1). MMP-1 was not selected because of overlap with MMP-8, while Granzyme A was added for personal interest. Due to the complexity in testing culture supernatant from QFT tube and the similar performance by testing supernatant without antigen stimulation (QFT NC tube), biomarker levels were tested in unstimulated plasma instead of culture supernatant to facilitate practical use. RANTES, MIG, IL-18, IL-22, MMP-8, and Granzyme A were included as cytokines for further testing in the 167 patients.

**TABLE 1:**
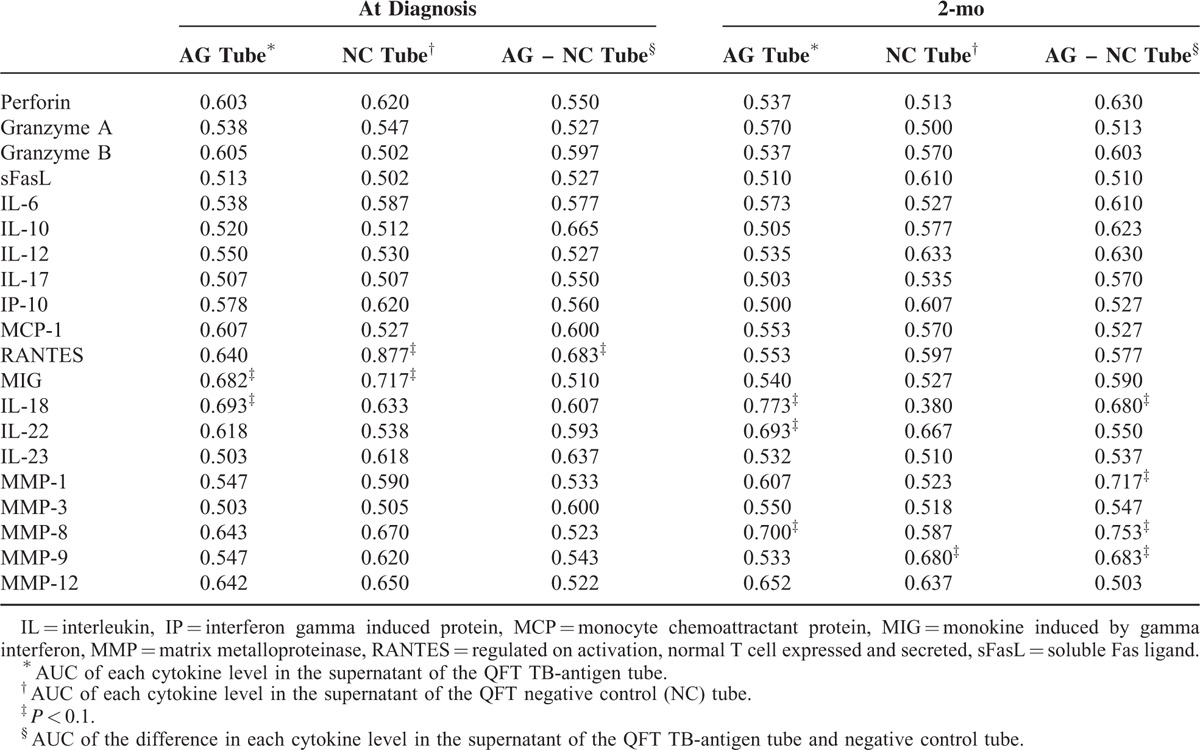
Area Under Receiver-Operating Characteristics Curve (AUC) of Each Cytokine Level in the Supernatant of QuantiFERON-TB Gold In-Tube Assay (QFT)

### Baseline Patient Characteristics

The demographic data of the 167 patients as regards their 2-month culture status (Table 2) revealed a mean age of 61.7 ± 18.4 years and male predominance (70.1%). More than half of patients were sputum acid-fast stain negative (55.1%). Diabetes mellitus (44 patients, 26.3%) and malignancy (29 patients, 17.4%) were 2 most common underlying diseases. In radiographic findings, fibrocalcified and cavitary lesions were seen in 129 (77.2%) and 22 (13.2%) patients, respectively.

**TABLE 2:**
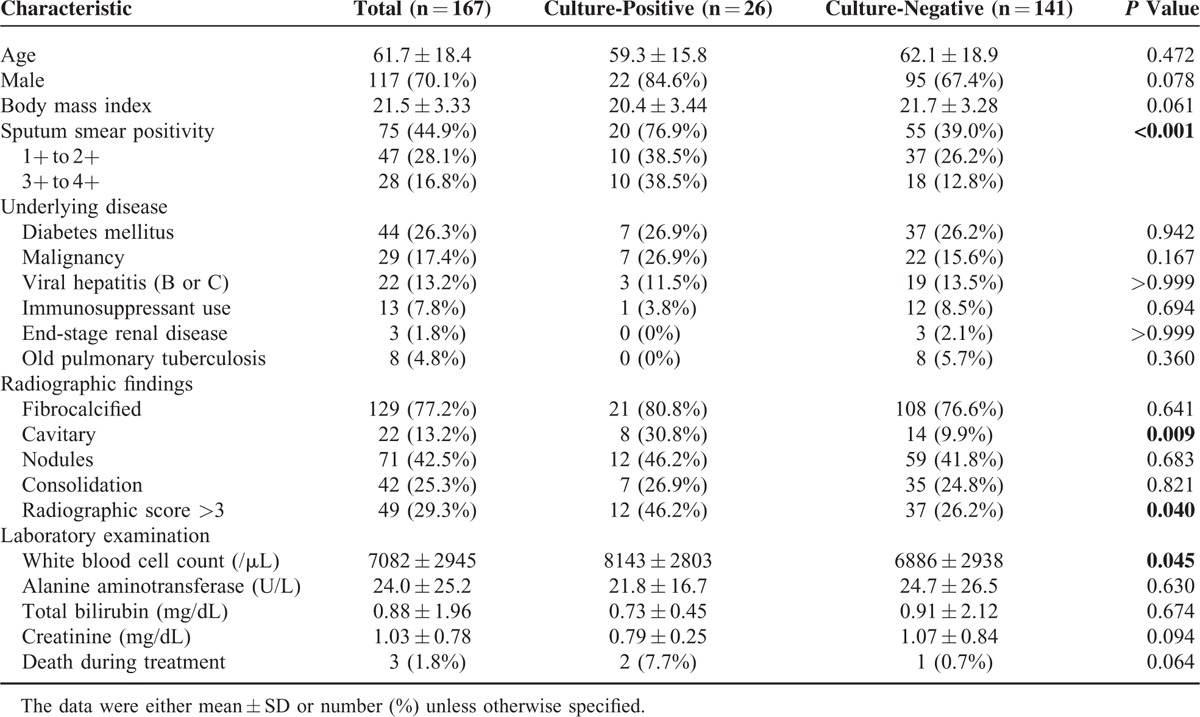
Clinical Characteristics of Patients Stratified by Culture Status of 2-Month Sputum

Persistent culture positivity was noted in 26 patients (15.6%). Compared with patients with 2-month culture conversion, these patients were significantly more like to be smear-positive cases (76.9% vs. 39%, *P* < 0.001), have cavitary lesions (30.8% vs. 9.9%, *P* = 0.009), and have radiographic score >3 (46.2% vs. 26.2%, *P* = 0.04).

### Cytokine Level at Diagnosis and 2-Month

**FIGURE 2:**
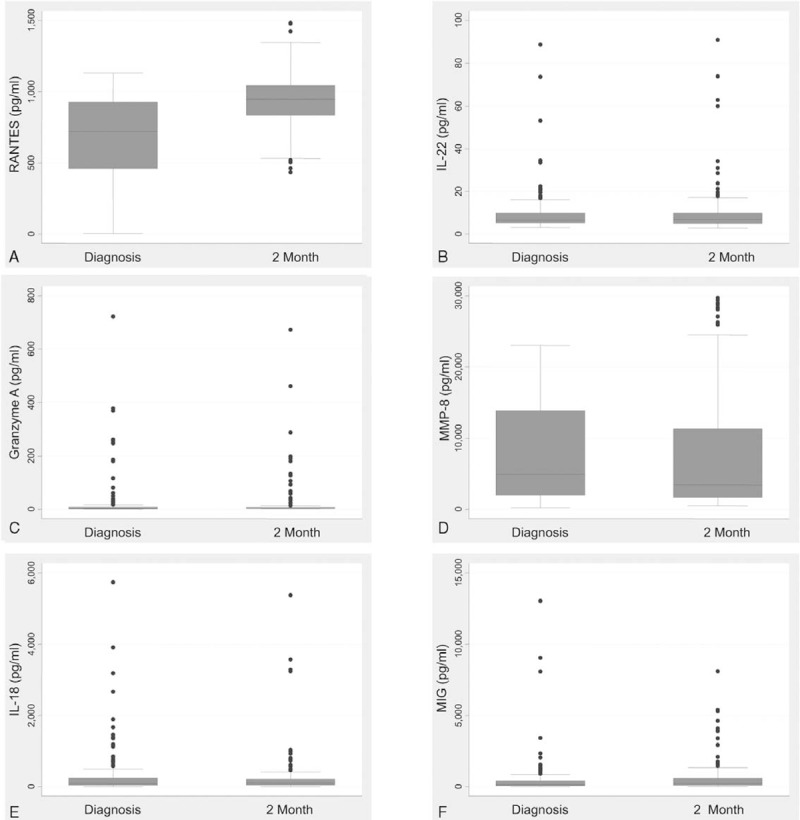
Box plots of plasma cytokine levels at diagnosis and at 2 months after the start of antituberculosis treatment. IL = interleukin, MIG = monokine induced by gamma interferon, MMP = matrix metalloproteinase, RANTES = regulated on activation, normal T cell expressed and secreted.

The levels of different cytokines at diagnosis and at 2 months after the start of treatment (Figure 2) revealed that RANTES level increased significantly after 2 months (694 ± 267 vs. 938 ± 182 pg/mL, *P* < 0.001). MIG level also showed an increasing trend but did not reach statistical significance (486 ± 1401 vs. 649 ± 1147 pg/mL, *P* = 0.069). Levels of IL-22, Granzyme A, MMP-8, IL-18, remained relatively unchanged after 2 months of anti-TB treatment.

The plasma biomarker levels were classified by radiographic score (>3 vs. ≤3) and bacterial load (sputum smear-negative vs. sputum smear-positive) (Table 3). Compared with patients with low radiographic score at diagnosis, those with high radiographic score at diagnosis had significantly lower plasma RANTES level at diagnosis (625 ± 287 vs. 723 ± 254 pg/mL, *P* = 0.03) and significantly higher MMP-8 level (9728 ± 7034 vs. 7285 ± 6750 pg/mL, *P* = 0.037) and MIG level (1119 ± 2452 vs. 223 ± 282 pg/mL, *P* = 0.014) at diagnosis. As regards sputum smear status, all 6 cytokine levels were not significantly different between smear-positive and smear-negative patients.

**TABLE 3:**
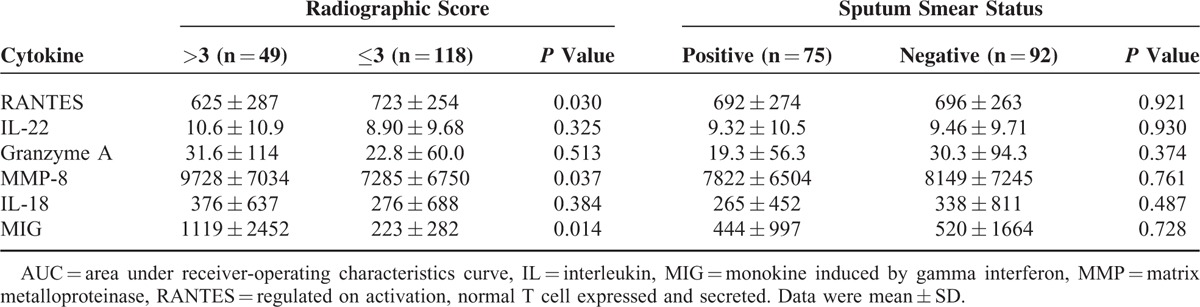
Plasma Cytokine Levels of Patients at Diagnosis Stratified by Radiographic Score and Sputum Smear Status

### Cytokine Level and 2-Month Culture Status

RANTES level at diagnosis (528 ± 227 vs. 725 ± 263 pg/mL, *P* < 0.001), Granzyme A at 2-month (8.0 ± 10.4 vs. 24.9 ± 78.2 pg/mL, *P* = 0.015), and MMP-8 level at 2-month (11539 ± 10515 pg/mL vs. 7064 ± 7943 pg/mL, *P* = 0.048) were significantly different between patients with persistent culture positivity and those with 2-month culture conversion (Table 4). In ROC analysis, both RANTES level at diagnosis (AUC: 0.725 [0.624–0.827], *P* < 0.001) and MMP-8 level at 2-month (AUC: 0.632 [0.512–0.753], *P* = 0.033) had a good performance in discriminating 2-month culture status.

**TABLE 4:**
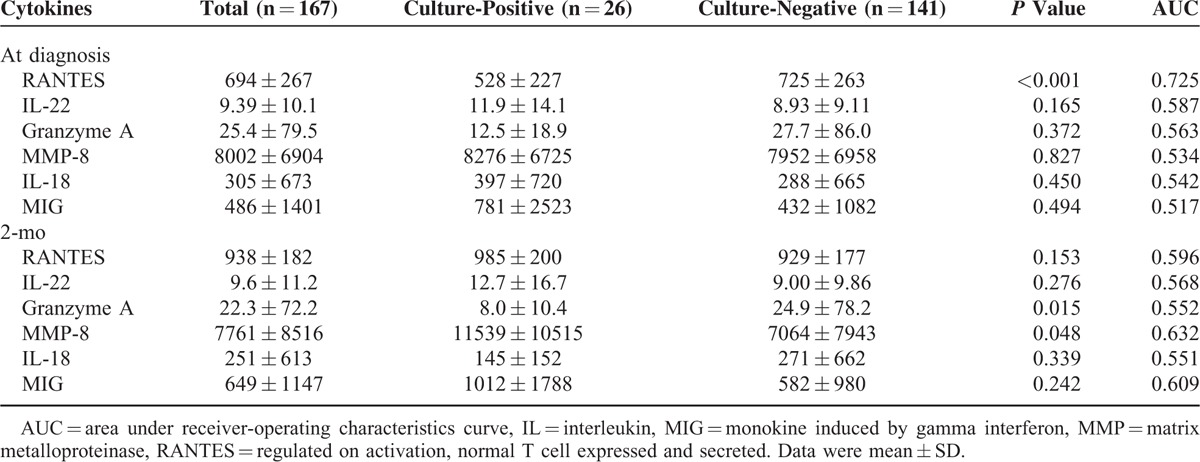
Plasma Cytokine Levels of Patients Stratified by Culture Status of 2-Month Sputum

The cut-off levels for RANTES at diagnosis and for MMP-8 at 2-month according to Youden's index were 440 and 3000 pg/mL, respectively. Logistic regression analysis, including demographic characteristics, radiographic findings, sputum mycobacteriology results, and plasma cytokine levels, revealed that low RANTES level at diagnosis (OR: 8.29 [3.03–22.6]), positive acid-fast smear at diagnosis (OR: 5.87 [2.01–17.1]), and high MMP-8 level at 2-month (OR: 3.36 [1.25–10.5]) were associated with persistent culture positivity at 2-month (Table 5).

**TABLE 5:**
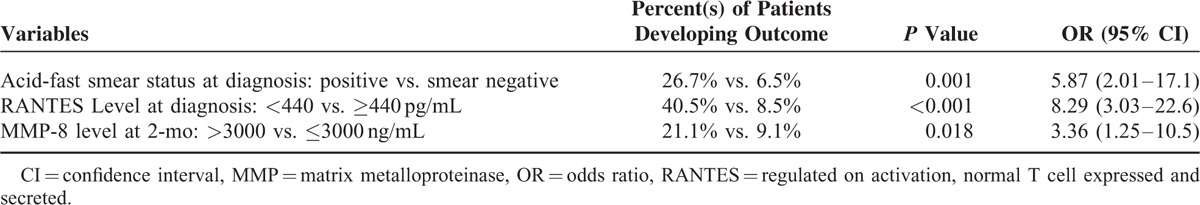
Factors Associated With Persistent Sputum Culture-Positivity at 2-Month, by Multivariable Logistic Regression Analysis

### Stratifying the Risk of Persistent Culture Positivity

**FIGURE 3:**
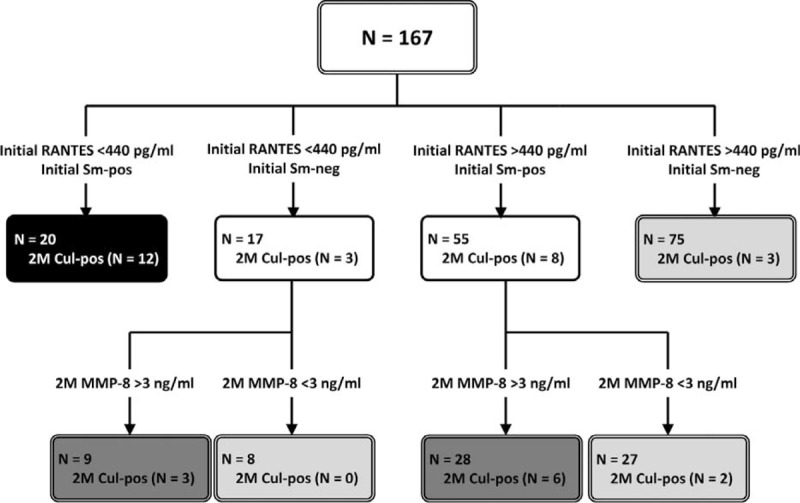
Risk assessment for persistent sputum culture positivity at 2 months after the start of antituberculosis treatment. Cul = mycobacterial culture, MMP = matrix metalloproteinase, Neg = negative, Pos = positive, RANTES = regulated on activation, normal T cell expressed and secreted, Sm = acid-fast smear.

The risk of persistent culture positivity was highest (80%) among patients with positive sputum acid-fast smear at diagnosis and plasma RANTES level of <440 pg/mL at diagnosis (Figure 3). In contrast, the risk was lowest (4%) among patients with negative sputum acid-fast smear and plasma RANTES level of ≥440 pg/mL at diagnosis.

Among the 72 patients with discrepant smear status and RANTES level at diagnosis, plasma MMP-8 at 2-month could aid in further risk stratification. For them, the risk of persistent culture positivity was 24% among the 37 patients with 2-month MMP-8 level >3000 pg/mL and 6% among the remaining 35.

## DISCUSSION

This study enrolled 167 culture-confirmed pulmonary TB patients and measured candidate cytokines at diagnosis and 2-month after the start of anti-TB treatment. The results showed that positive sputum smear at diagnosis, plasma RANTES level of <440 pg/mL at diagnosis, and MMP-8 level of >3000 pg/mL at 2-month were associated with 2-month culture persistence. More than one-third of patients with any 2 of the 3 risk factors had positive culture at 2-month. The risk was even higher in those with positive sputum acid-fast smear and low plasma RANTES level at diagnosis. In contrast, persistent culture-positive was unlikely among patients with negative sputum acid-fast smear and high plasma RANTES level at diagnosis. For other patients, MMP-8 level at 2-month helped stratify risk for persistent culture positivity.

RANTES (CCL5) is a member of the chemokine family, which is considered to be involved in leukocyte migration.^27^ It acts as a proinflammatory mediator and is associated with various inflammatory diseases such as atherosclerosis, asthma, fibrosis, and transplant rejection.^27^ In TB, RANTES has a protective role in *M*. *tuberculosis* infection by forming granuloma, limiting pathogen growth, and preventing lung tissue damage.^28^

Wolday et al^29^ evaluated RANTES level in 21 HIV-and-TB coinfected patients at time the point of diagnosis and 2-month after the start of treatment and showed an increasing trend from diagnosis to 2-month, but not reaching statistical significance. In another study, RANTES level was not significantly different between patients with active pulmonary TB (N = 23) and BCG-vaccinated healthy controls (N = 30).^30^ Like a previous study on 28 African pulmonary TB patients showing that RANTES level correlated with the speed of sputum conversion,^31^ the presents study demonstrated that a higher RANTES level was associated with better therapeutic response as defined by culture conversion on the 2nd month of treatment.

Traditionally, we can only rely on initial grading of sputum acid-fast smear to predict 2-month culture status. In this study, positive initial sputum acid-fast smear has a positive predictive value of only 27% (20/75). However, if initial RANTES status is added into the prediction model, the combined lower RANTES level and smear positivity has a positive predictive value of 60% (12/20). Among patients with both high RANTES level and smear negativity, 96% (72/75) will have culture conversion at 2 months. This means that by combining RANTES level and sputum smear status at diagnosis, only a small proportion of patients will be misclassified based on the 2-month culture status.

MMPs (matrix metalloproteinase) are a family of zinc-containing proteases not only responsible for extracellular matrix degradation but also for the direct activation of various signaling molecules.^24^ In pulmonary TB patients, MMPs are responsible for cavity formation, erosion of granuloma into the bronchus, and the dissemination of *M. tuberculosis*.^24^ MMP-8, also known as collagenase-2 or neutrophil collagenase, is involved in various lung diseases such as asthma, bronchiectasis, pulmonary emphysema, acute lung injury, and pulmonary TB.^24,32,33^ As shown in this study, the beneficial role of MMP-8 in risk stratification may be related to the fact that a higher MMP-8 level indicates greater disease burden and severity.

This study has several limitations. First, most of the patients were relatively old and with higher prevalence of comorbidities compared with previous studies.^8^ This study was also conducted in a tertiary referral center in a country with medium TB incidence.^16,34,35^ Nonetheless, the patients are more similar to those encountered by physicians in developed countries. Second, the findings were not validated in a second cohort. Given the heterogeneity and even ethnic variation of TB patient populations, the best validation cohort would be conducted by other research groups in different regions. Last, treatment outcomes other than 2-month culture conversion were not investigated. The lengthy follow-up period and relatively small proportion of patients in this study preclude the use of treatment failure and TB relapse as outcomes.^2,36^

In conclusion, together with initial smear status, plasma RANTES level (cut-off value 440 pg/mL) can better predict culture status at 2-month of anti-TB treatment. Among patients with either low RANTES/smear negativity or high RANTES/smear positivity at diagnosis, plasma MMP-8 level (cut-off value 3000 pg/mL) at 2-month can also aid in differentiating culture status at 2 months of treatment. With more accurate prediction of treatment response, patient management can be optimized, ensuring better outcomes.
